# Anti-inflammatory effect of ethanolic extract from *Myagropsis myagroides* on murine macrophages and mouse ear edema

**DOI:** 10.1186/1472-6882-12-171

**Published:** 2012-10-03

**Authors:** Eun-Ji Joung, Min-Sup Lee, Ji-Woong Choi, Jong-Soon Kim, Taisun Shin, Bok-Mi Jung, Na Young Yoon, Chi-Won Lim, Jae-Il Kim, Hyeung-Rak Kim

**Affiliations:** 1 Department of Food Science and Nutrition, Pukyong National University, Busan, 608-737, South Korea; 2 Department of Food Science and Nutrition, Chonnam National University, Yeosu, 550-749, South Korea; 3 Food and Safety Research Division, National Fisheries Research and Development Institute, Gijang-gun, Busan, 619-705, Korea

**Keywords:** Anti-inflammation, *Myagropsis myagroides*, MAPK, NF-κB, RAW 264.7 cells

## Abstract

**Background:**

This study aims to investigate anti-inflammatory effect of ethanolic extract of *Myagropsis myagroides* (EMM) in the lipopolysaccharide (LPS)-stimulated RAW 264.7 macrophages and the phorbol 12-myristate 13-acetate (PMA)-induced ear edema in mice, and to clarify its underlying molecular mechanisms.

**Methods:**

The levels of nitric oxide (NO), prostaglandin E_2_ (PGE_2_), and pro-inflammatory cytokines were measured by Griess assay and enzyme linked immunosorbent assay. The expressions of inducible nitric oxide synthase (iNOS), cyclooxygenase-2 (COX-2), mitogen-activated protein kinases (MAPKs), and Akt were measured using Western blotting. Nuclear translocation and transcriptional activation of nuclear factor-κB (NF-κB) were determined by immunocytochemistry and reporter gene assay, respectively. PMA-induced mouse ear edema was used as the animal model of inflammation. Anti-inflammatory compounds in EMM were isolated using high-performance liquid chromatography and identified by nuclear magnetic resonance.

**Results:**

EMM significantly inhibited the production of NO, PGE_2_, and pro-inflammatory cytokines in a dose-dependent manner and suppressed the expression of iNOS and COX-2 in LPS-stimulated RAW 264.7 cells. EMM strongly suppressed nuclear translocation of NF-κB by preventing degradation of inhibitor of κB-α as well as by inhibiting phosphorylation of Akt and MAPKs. EMM reduced ear edema in PMA-induced mice. One of the anti-inflammatory compounds in EMM was identified as 6,6’-bieckol.

**Conclusions:**

These results suggest that the anti-inflammatory properties of EMM are associated with the down-regulation of iNOS, COX-2, and pro-inflammatory cytokines through the inhibition of NF-κB pathway in LPS-stimulated macrophages.

## Background

Inflammation is a complex response of host defense against microbial infection, endotoxin exposure, or cell injury, and ultimately leads to the restoration of normal cell structure and function. Macrophages are known to play a key role in host defense mechanism, and are activated by exposure to interferon-γ, pro-inflammatory cytokines, and bacterial lipopolysaccharides (LPSs) [1,2]. Activated macrophages play a pivotal role in inflammatory diseases by excessive production of pro-inflammatory cytokines, including tumor necrosis factor (TNF)-α, interleukin (IL)-1β, and IL-6, and inflammatory mediators such as nitric oxide (NO) and prostaglandin E_2_ (PGE_2_) [3,4]. Expressions of these cytokines and mediators are regulated by the activation of nuclear factor-kappaB (NF-κB), which is critically involved in the pathogenesis of rheumatism and other chronic inflammatory diseases [5]. In the unstimulated condition, NF-κB is located in the cytoplasm as an inactive complex bound to inhibitor of kappaB and (IκB)-α. Treatment of LPS activates the IκB-α kinase (IKK) complex, resulting in the phosphorylation, ubiquitination, and degradation of IκB-α, inducing the translocation of NF-κB into the nucleus for transcriptional activation. The activation of NF-κB is also regulated by cellular kinases such as mitogen-activated protein kinases (MAPKs) and Akt [6,7]. Hence, substances which inhibit the activation of NF-κB are considered as potential anti-inflammatory agents.

*Myagropsis myagroides* is one of the most abundant brown algae, which has been used as a part of the diet and folk medicines in Korea, Japan, and China for centuries. This species contains high level of phlorotannins and pigments. Recently, phlorotannins have been reported to have several biological activities, such as antioxidation [8], antidiabetic complications [9], antiamnesia [10], anti-inflammation [11], and hepatoprotection [12]. As a biological activity of *M. myagroides,* previous study revealed a cytoprotective effect on carbon tetrachloride-induced hepatotoxicity by its antioxidant properties [13]. Recently, fucoxanthin isolated from *M. myagroides* inhibited the production of inflammatory mediators and pro-inflammatory cytokines via inhibiting NF-κB activation in RAW 264.7 cells [14]. However, we found an anti-inflammatory activity in the ethanolic extract of sun-dried *M. myagroides* (EMM) in LPS-stimulated RAW 264.7 cells. Since fucoxanthin prone to rapidly oxidized during sun-drying due to instability under oxygen and sun light exposure, we hypothesized that other anti-inflammatory compounds might be exist in EMM. Furthermore, we confirmed that EMM does not contain any detectable amount of fucoxanthin by HPLC chromatographic analysis at 410 nm. To our knowledge, no previous study has been reported on the anti-inflammatory activity of *M. myagroides* except fucoxanthin. Therefore, we investigated the anti-inflammatory and anti-edema properties of EMM and its underlying mechanisms using cultured RAW 264.7 cells and identified anti-inflammatory compound in EMM.

## Methods

### Chemicals

CellTiter96^®^ AQ_ueous_ One Solution Cell Proliferation assay kit, luciferase assay kit, murine NF-κB promoter/luciferase DNA, pRL-TK DNA and reverse transcriptase were obtained from Promega (Madison, WI, USA). Primary antibodies and secondary antibodies were purchased from Santa Cruz Biotechnology (Santa Cruz, CA, USA). Enhanced chemiluminescence (ECL) detection kit was obtained from GE Healthcare Bio-Science (Piscataway, NJ, USA). Dulbecco’s modified Eagle's medium (DMEM), penicillin-streptomycin mixture, 0.25% trypsin-ethylenediaminetetraacetic acid (EDTA), and fetal bovine serum (FBS) were purchased from Gibco BRL Life Technologies (Grand Island, NY, USA). 4’,6-Diamidino-2-phenylindole (DAPI), Alexa Fluor® 488-conjugated secondary antibody, Lipofectamine/Plus, and TRIzol were purchased from Invitrogen (Carlsbad, CA, USA). LPS (*Escherichia coli* O55:B5), bovine serum albumin (BSA), 2',7'-dichlorofluorescein diacetate (DCF-DA), dimethyl sulfoxide (DMSO), indomethacin (Indo), and phorbol 12-myristate 13-acetate (PMA) were purchased from Sigma-Aldrich Corporation (St. Louis, MO, USA). Specific kinase inhibitors (Wortmannin, PD98059, SP600125, and SB203580) were purchased from Abcam (Cambridge, UK).

### Preparation of EMM

*M. myagroides was* harvested from the coast of Busan, South Korea, in April 2010. Taxonomic identification was confirmed by an agal taxonomist (C.K Choi), at the Department of Ecological Engineering, Pukyong National University, Korea. The seaweed was rinsed in tap water to remove salt, and air-dried under sunshine for 3 days. A dried sample was ground with a hammer mill and the powder was stored at −20°C until used. The dried powder (500 g) of *M. myagroides* was extracted three times with 96% ethanol (3 L/each) for 3 h at 70°C. The combined extracts were filtered and concentrated using rotary vacuum evaporator (Tokyo Eyela, Tokyo, Japan) at 40°C and freeze-dried EMM (89.7 g) was stored in freezer (−70°C) until used.

### Cell culture and viability assay

Murine macrophage RAW 264.7 cells (ATCC, Rockville, MD, USA) were cultured at 37°C in DMEM supplemented with 10% FBS, penicillin (100 units/mL), and streptomycin sulfate (100 μg/mL). Cell viability was determined by 3-(4,5-dimethylthiazol-2-yl)-5-(3-carboxymethoxyphenyl)-2-(4-sulfophenyl)-2H-tetrazolium (MTS) assay using a CellTiter96^®^ AQ_ueous_ One Solution Cell Proliferation assay kit according to the manufacturer’s instruction. Briefly, cells were inoculated at a density of 5 × 10^5^ cells/well into 96-well plate and cultured at 37°C for 24 h. The culture medium was replaced with 200 μL of serial dilutions of EMM, and the cells incubated for 24 h. The medium was replaced with 95 μL fresh medium and 5 μL MTS solution. After 1 h, the absorbance at 490 nm was measured using a microplate reader (Glomax Multi Detection System, Promega).

### Measurement of NO, PGE_2_, TNF-α, IL-1α and IL-6

RAW 264.7 cells were plated in a 12-well plate at a density of 1 × 10^6^ cells/well and incubated for 24 h. Cultured cells were treated with various concentrations of EMM for 1 h, and then stimulated with 1 μg/mL LPS for 24 h. Cultured media were collected after centrifugation at 2,000*g* for 10 min and stored at −70°C until tested. The nitrite concentration in the cultured media was measured as an indicator of NO production, according to the Griess reaction [15]. Levels of PGE_2_, IL-1β, IL-6, and TNF-α in cultured media were quantitated by enzyme-linked immunosorbent assay (ELISA, R&D Systems, Minneapolis, MN, USA) according to the manufacturer’s instructions.

### Reverse transcription-polymerase chain reaction (RT-PCR)

RAW 264.7 cells placed in a 6-well plate were pretreated with EMM for 1 h and then stimulated with LPS for 6 h. Total RNA from each group was isolated with the TRIzol reagent. Five microgram of total RNA was used for reverse transcription using oligo-dT-adaptor primer and superscript reverse transcriptase. PCR was carried out with the gene-specific primers: COX-2 sense, 5’-CAGCAAATCCTTGCTGTTCC-3’; COX-2 antisense, 5’-TGGGCAAAGAATGCAAACAT-3’; iNOS sense, 5’-CACCTTGGAGTTCACCCAGT-3’; iNOS antisense, 5’-ACCACTCGTACTTGGGATGC-3’; glyceraldehyde 3-phosphate dehydrogenase (GAPDH) sense, 5’-GACCCCTTCATTGACCTCAA-3’; GAPDH antisense, 5’-CTTCTCCATGGTGGTGAAGA-3’. GAPDH was used as an internal standard to evaluate relative expression of COX-2 and iNOS. Densitometric analysis of the data obtained from at least three independent experiments was performed using cooled CCD camera system EZ-Capture II and CS analyzer ver. 3.00 software (ATTO & Rise Co., Tokyo, Japan).

### Transient transfection and luciferase assay

Murine NF-κB promoter/luciferase DNA (1 μg) along with 20 ng control pRL-TK DNA was transiently transfected into 2 × 10^5^ RAW 264.7 cells/well in a 24-well plate using Lipofectamine/Plus reagents for 40 h. Cells pretreated with 0–100 μg/mL EMM for 1 h were stimulated with LPS (1 μg/mL) for 6 h. Each well was washed twice with cold phosphate-buffered saline (PBS), harvested in 100 μL of lysis buffer (0.5 mM HEPES, pH 7.8, 1% Triton N-101, 1 mM CaCl_2_, and 1 mM MgCl_2_) and used for the measurement of luciferase activity using a luciferase assay kit. Luminescence was measured on a TopCount microplate scintillation and luminescence counter (PerkinElmer, Santa Clara, CA, USA) in single-photon counting mode for 0.1 min/well, following a 5 min adaptation in the dark. Luciferase activity was normalized to the expression of control pRL-TK.

### Preparation of cytosolic and nuclear extracts

RAW 264.7 cells (5 × 10^6^ cells/well ) pretreated with EMM for 1 h were stimulated with LPS for 0.5 h. Cells were washed twice with cold PBS and harvested. Cell pellets were resuspended in 300 μL of hypotonic buffer [10 mM HEPES/KOH, 10 mM KCl, 2 mM MgCl_2_, 0.1 mM EDTA, 1 mM DTT, and 0.5 mM phenylmethylsulfonyl fluoride (PMSF), pH 7.9] and incubated on ice for 15 min. After vortexing for 10 s, homogenates were separated into supernatants (cytoplasmic compartments) and pellets (nuclear components) by centrifugation at 13,000*g* for 10 min. The pellet was gently resuspended in 40 μL complete lysis buffer (50 mM HEPES/KOH, 50 mM KCl, 1 mM DTT, 300 mM NaCl, 1% IGEPAL CA-630, 0.1 mM EDTA, 10% glycerol, and 0.5 mM PMSF, pH 7.9) and centrifuged at 13,000*g* for 20 min at 4°C. The supernatant was used as the nuclear extract.

### Western immunoblot analysis

RAW 264.7 cells were incubated with various concentrations of EMM for 1 h and stimulated with LPS (1 μg/mL) for 30 min. RAW 264.7 cells were washed twice with cold PBS and lysed with lysis buffer (50 mM Tris–HCl, pH 7.5, 150 mM NaCl, 1% IGEPAL CA-630, 1% Tween 20, 0.1% SDS, 1 mM Na_3_VO_4_, 10 μg/mL leupeptin, 50 mM NaF, and 1 mM PMSF) on ice for 1 h. After centrifugation at 18,000*g* for 10 min, the protein concentrations in the supernatants were determined, and aliquots of the protein (40 μg) were separated by sodium dodecyl sulfate-polyacrylamide gel electrophoresis (SDS-PAGE) and transferred onto a nitrocellulose membrane. The membrane was blocked with 5% nonfat dry milk in Tris-buffered saline with 0.1% Tween 20 (TBST) for 1 h, followed by the incubation for 2 h with primary antibody in TBST containing 5% nonfat dry milk. The blots were treated with horseradish peroxidase-conjugated secondary antibody in TBST containing 5% nonfat dry milk for 1 h, and immune complexes were detected using an ECL detection kit. Densitometric analysis of the data obtained from at least three independent experiments was performed using cooled CCD camera system EZ-Capture II and CS analyzer ver. 3.00 software.

### Immunofluorescence analysis

RAW 264.7 cells were maintained on glass coverslips (SPL Lifesciences Co., Gyeonggi-do, Korea) to analyze nuclear localization of NF-κB in 24-well plates for 24 h. Cells treated with EMM for 1 h were incubated with LPS (1 μg/mL) for 30 min as described in Lee et al. [16]. Cells were fixed in 4.0% paraformaldehyde in PBS for 15 min at room temperature, and then permeabilized with 0.5% Triton X-100 in PBS for 10 min. Cells were washed with PBS and blocked with 3% BSA/PBS for 30 min. Thereafter, cells were incubated with an anti-NF-κB polyclonal antibody diluted in 3% BSA/PBS for 2 h, and incubated with Alexa Fluor® 488-conjugated secondary antibody diluted in 3% BSA/PBS for 1 h. Cells were stained with 2 μg/mL DAPI and images were captured using an LSM700 laser scanning confocal microscope (Carl Zeiss, Oberkochen, Germany).

### Mouse model and PMA-induced ear edema

Animal studies were conducted after the experimental protocols and procedures were approved by the Animal Ethics Committee of the Pukyong National University. ICR mice (male, 25–30 g) were purchased from the Samtako Bio Korea Co. (Gyeonggido, Korea) and permitted free access to a standard chow diet and tap water. All mice were acclimatized for 1 week prior to the experiments and maintained at 22 ± 2°C with a relative humidity of 50 ± 5% and 12 h light–dark cycle. Ear edema was induced on the right ear of mouse with PMA according to Garrido et al. [16]. Briefly, the control group received normal saline, and the other three groups include a PMA alone, PMA + Indo (a positive control), and EMM administered groups (PMA + EMM). The left ear (reference) received the vehicle (30 μL acetone). PMA (6 μg/ear in 30 μL acetone) was applied to the inner surface of the right ear of ICR mouse. EMM (90 μg per ear in 30 μL acetone) or Indo (1 mg/ear in 30 μL acetone) was topically administered 1 h before PMA application. Six hours after PMA application, mice were killed by cervical dislocation and a 6 mm diameter disc from each ear was removed with a metal punch and weighed. Ear edema weight was calculated by subtracting the weight of the left ear from the right ear (treatment). Inhibition percentage (IP) was expressed as a reduction in weight compared to the PMA-treated group.

### Isolation of phlorotannins from EMM

Aliquots of EMM were separated by Shimadzu high-performance liquid chromatography (HPLC) system with Luna RP-18 [Luna C18(2), 5 μm, 250 × 10 mm, Phenomenex, Torrence, CA, USA]. The separation of EMM was conducted using the mobile phase of 0.1% formic acid in water (solvent A) and 0.1% formic acid in acetonitrile (solvent B). The elution profile consisted of a linear gradient from 20 to 100% solvent B for 110 min and hold for 15 min and then re-equilibration of the column with 20% solvent B for 15 min. The flow rate was 0.34 mL/min at 35°C oven temperature and detection was performed at 270 nm. The purity was determined by HPLC (purity: >99%) and the chemical structure of the isolated compounds were identified as eckol [17] and 6,6’-bieckol [8,12] by comparing their ^1^H- and ^13^C-NMR data.

### Statistical analysis

Data were expressed as the means ± standard deviations (SDs) from at least three separate experiments unless otherwise indicated. Data were analyzed using one-way analysis of variance. Differences were considered significant at values of *p* < 0.05. All analyses were performed using SPSS for Windows, version 10.07 (SPSS Inc., Chicago, IL, USA).

## Results

### Effect of EMM on LPS-induced NO and PGE_2_ production

**Figure 1:**
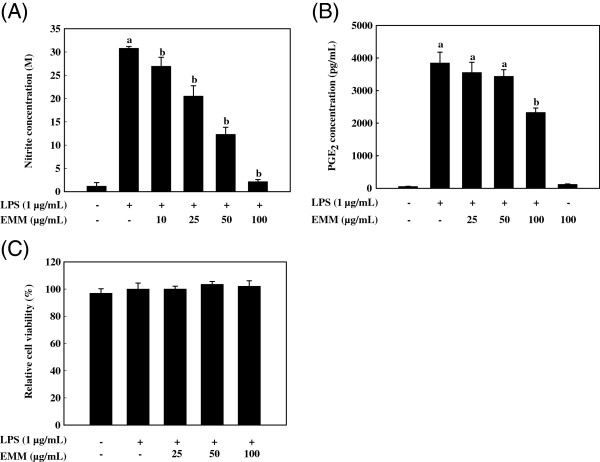
**Effect of EMM on LPS-induced NO and PGE**_**2**_**production in RAW 264.7 cells.** Cells pretreated with different concentrations of EMM for 1 h were stimulated with LPS (1 μg/mL) for 24 h. The treated culture media were used to measure the amount of NO production (**A**) and PGE_2_ production (**B**). Cytotoxic effect of EMM was measured by MTS assay (**C**). Values are the means ± SDs of three independent experiments. ^a^*p* < 0.05 indicates significant differences compared to the control group. ^b^*p* < 0.05 indicates significant differences compared to the LPS-only treated group.

NO production, measured as nitrite, was increased by LPS treatment; however, EMM significantly reduced NO levels in LPS-stimulated cells in a dose-dependent manner (*p* < 0.05, Figure 1A). Increased PGE_2_ production by LPS was also significantly suppressed by 100 μg/mL EMM in RAW 264.7 cells (Figure 1B). To exclude the possibility that the decreased NO and PGE_2_ levels were due to cell death, cytotoxicity of EMM was determined by MTS assay. The result demonstrated that EMM showed no cytotoxicity in RAW 264.7 cells up to 100 μg/mL (Figure 1C). Thus, the inhibitory effects of EMM on NO and PGE_2_ production were not due to cytotoxicity.

### Effect of EMM on LPS-induced iNOS and COX-2 proteins and mRNA expression

**Figure 2:**
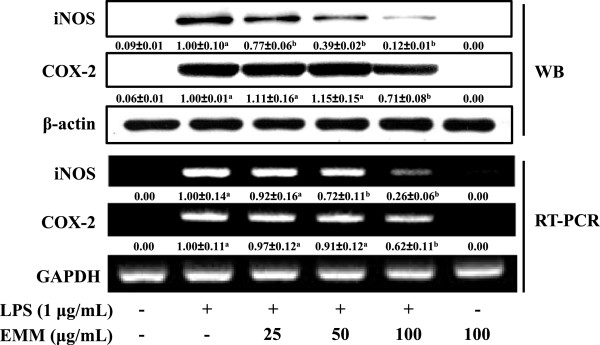
**Effect of EMM on LPS-induced iNOS and COX-2 protein and mRNA expression in RAW 264.7 cells.** (**A**) Cells were pretreated with indicated concentration of EMM for 1 h and stimulated with LPS (1 μg/mL) for 16 h. Forty microgram of proteins were subjected to 10% SDS-PAGE. The expression of iNOS, COX-2, and β-actin protein was detected by Western blotting using corresponding antibodies. (**B**) Cells were pretreated with EMM for 1 h and stimulated with LPS for 6 h, and then total RNA was prepared for RT-PCR. The results presented are representative of three independent experiments. ^a^*p* < 0.05 indicates significant differences compared to the control group. ^b^*p* < 0.05 indicates significant differences compared to the LPS-only treated group.

As shown in Figure 2, EMM strongly inhibited iNOS protein production in a dose-dependent manner; however, COX-2 protein production was only inhibited by 100 μg/mL EMM, which is similar pattern observed in the PGE_2_ secretion (Figure 1B). In addition to iNOS protein, EMM also inhibited iNOS mRNA expression in a dose-dependent manner. Like inhibition of COX-2 protein, COX-2 mRNA expression was partially inhibited by 100 μg/mL EMM. These results suggest that the EMM-mediated inhibition of NO and PGE_2_ production in LPS-stimulated macrophages is associated with downregulation of iNOS and COX-2 expression at transcriptional level.

### Effect of EMM on LPS-induced pro-inflammatory cytokines

**Figure 3:**
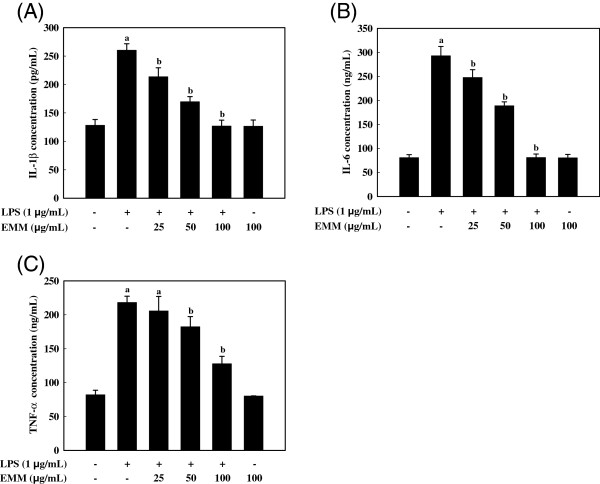
**Effects of EMM on pro-inflammatory cytokine productions in LPS-stimulated RAW 264.7 cells.** Cells were treated with various concentrations of EMM for 1 h, and then stimulated with LPS for 24 h. IL-1β (**A**), IL-6 (**B**), and TNF-α (**C**) in the cultured media were measured by ELISA. Data are means ± SDs of three independent experiments. ^a^*p* < 0.05 indicates significant differences compared to the control group. ^b^*p* < 0.05 indicates significant differences compared to the LPS-only treated group.

Increased levels of IL-1β (Figure 3A), IL-6 (Figure 3B), and TNF-α (Figure 3C) in RAW 264.7 cells by LPS stimulation were dramatically reduced in a dose-dependent manner by exposure to EMM (*p* < 0.05). This result indicates that EMM efficiently suppressed LPS-induced IL-1β, IL-6, and TNF-α release, which supports the hypothesis that EMM inhibits the initial phase of the LPS-stimulated inflammatory response.

### Effect of EMM on LPS-induced activation and translocation of NF-κB

**Figure 4:**
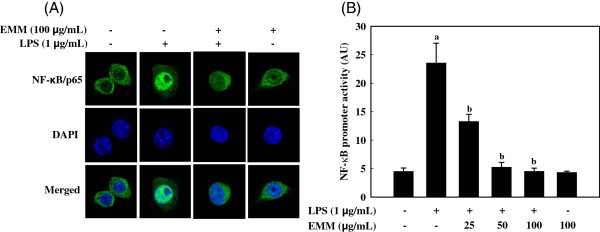
**Effect of EMM on activation and translocation of NF-κB in RAW 264.7 cells.** (**A**) Cells were pretreated with and without EMM for 1 h followed by LPS stimulation for 30 min. NF-κB/p65 subunits were probed by anti-NF-κB polyclonal primary antibody and Alexa Fluor® 488-conjugated secondary antibody. The nuclei were stained with DAPI and the images were captured by confocal microscopy. (**B**) Cells transfected with NF-κB promoter-containing luciferase DNA were pretreated with various concentrations of EMM for 1 h followed by LPS stimulation for 6 h. ^a^*p* < 0.05 indicates significant differences compared to the control group. ^b^*p* < 0.05 indicates significant differences compared to the LPS-only treated group.

Confocal microscopy revealed that NF-κB/p65 was distributed mostly in the cytoplasm in unstimulated cells. After stimulation with LPS, most cytoplasmic NF-κB/p65 were translocated into the nucleus, as shown by strong NF-κB/p65 intensity in the nucleus (Figure 4A). The level of p65 in the nucleus was markedly reduced by treatment with EMM. Considering the inhibitory effects of EMM on LPS-induced NF-κB translocation into nucleus, we determined the effect of EMM on the promoter activity of NF-κB in LPS-stimulated cells. Data suggested that EMM treatment significantly inhibited LPS-induced NF-κB promoter activity (Figure 4B, *p* < 0.05).

**Figure 5:**
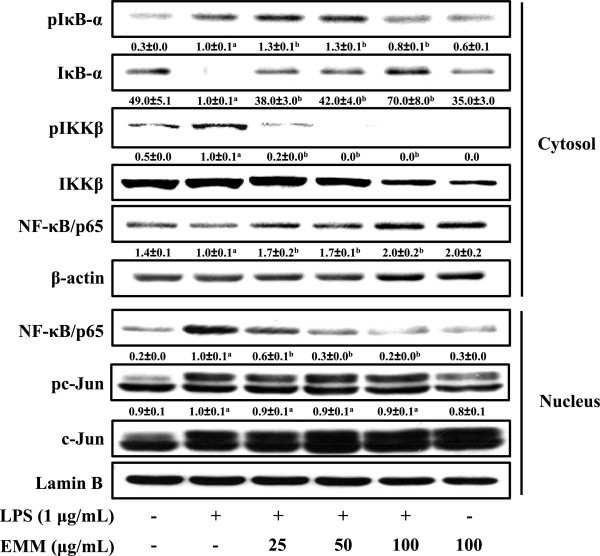
**Inhibitory effect of EMM on the degradation of IκB-α and the activation of NF-κB in LPS-stimulated RAW 264.7 cells.** Cells were incubated with various concentrations of EMM for 1 h, and then stimulated with LPS (1 μg/mL) for 30 min. Cytosolic and nuclear fractions were prepared and analyzed by Western blotting using corresponding antibodies. The results presented are representative of three independent experiments. ^a^*p* < 0.05 indicates significant differences compared to the control group. ^b^*p* < 0.05 indicates significant differences compared to the LPS-only treated group.

LPS treatment resulted in increased IKKβ phosphorylation and IκB-α degradation compared to non-treated control group, and EMM treatment suppressed IKKβ phosphorylation and IκB-α degradation, recovered the control level of cytosolic IκB-α in a dose-dependent manner (Figure 5). As a result of IκB-α degradation, the increased NF-κB level in nucleus after LPS stimulation was reduced by EMM treatment in a dose-dependent manner (Figure 5). However, phosphorylation of c-Jun, a component of transcription factor AP-1, was not reduced by EMM treatment. These results indicate that the EMM-mediated inhibition of iNOS, COX-2, and pro-inflammatory cytokines production was mainly regulated by the transcription factor NF-κB in LPS-stimulated macrophages.

### Effect of EMM on LPS-induced phosphorylation of MAPKs and Akt

**Figure 6:**
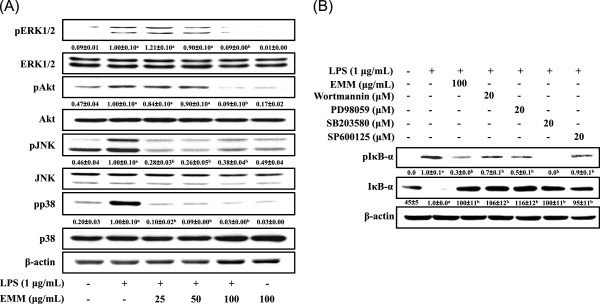
**Effect of EMM on the phosphorylations of MAPKs and Akt in RAW 264.7 cells.** (**A**) Cells pretreated with indicated concentrations of EMM for 1 h were stimulated with LPS (1 μg/mL) for 30 min. (**B**) Cells pretreated with indicated concentrations of EMM or inhibitors for 1 h were stimulated with LPS (1 μg/mL) for 30 min. Whole cell lysates (40 μg) were analyzed by Western blotting using corresponding antibodies. The results presented are representative of three independent experiments. ^a^*p* < 0.05 indicates significant differences compared to the control group. ^b^*p* < 0.05 indicates significant differences compared to the LPS-only treated group.

As shown in Figure 6A, EMM inhibited phosphorylation of JNK, p38 MAPK, ERK, and Akt induced by LPS in RAW 264.7 cells, suggesting the additional characteristics of EMM to regulate NF-κB pathway via blocking the phosphorylation of MAPKs and Akt proteins in response to LPS signal. To further confirm the association of these signaling molecules with the EMM’s anti-inflammatory effect, IκB-α phosphorylations in LPS-stimulated RAW 264.7 cells were measured in the presence of PI3K/Akt pathway inhibitor (Wortmannin), ERK inhibitor (PD98059), JNK inhibitor (SP600125), or p38 inhibitor (SB203580). As shown in Figure 6B, IκB-α phosphorylation was remarkably suppressed by p38 inhibitor, which is consistent with the result of Figure 6A. Other inhibitors such as Wortmannin, PD98059, and SP600125, moderately inhibited the phosphorylation of IκB-α. These data confirm additional characteristics of EMM on the phosphorylation of MAPKs and Akt proteins for NF-κB activation in response to LPS.

### Effect of EMM on PMA-induced ear edema in mouse

**Figure 7:**
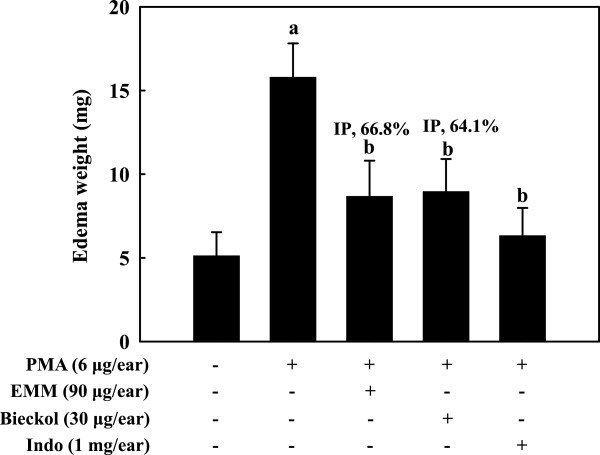
**Effect of EMM on ear edema induced by PMA in mouse.** EMM, 6,6’-bieckol or indomethacin (Indo) was administered topically on inner surface of the right ears of mice 1 h before application of PMA. Six hours after application of PMA, ear edema and the inhibition percentage (IP) of the ear edema by the treatment were calculated. ^a^*p* < 0.05 indicates significant differences compared to the non-treated group. ^b^*p* < 0.05 indicates significant differences compared to the PMA-only treated group.

Ear edema was induced by the application of PMA to the mouse ears. As shown in Figure 7, the application of PMA for 6 h significantly increased the weight of ear edema. Indo, a common clinical non-steroidal anti-inflammatory drug, was used as positive control for the inhibitory effect on the ear edema. As shown in Figure 7, pre-treatment of 1 mg/ear of Indo could effectively reduce ear edema after 6 h PMA stimulation (*p* < 0.05). Similarly, pre-administration of EMM (90 μg/ear) markedly inhibited the PMA-induced ear edema with 66.8% inhibition (*p* < 0.05).

### Isolation of anti-inflammatory compounds from EMM

**Figure 8:**
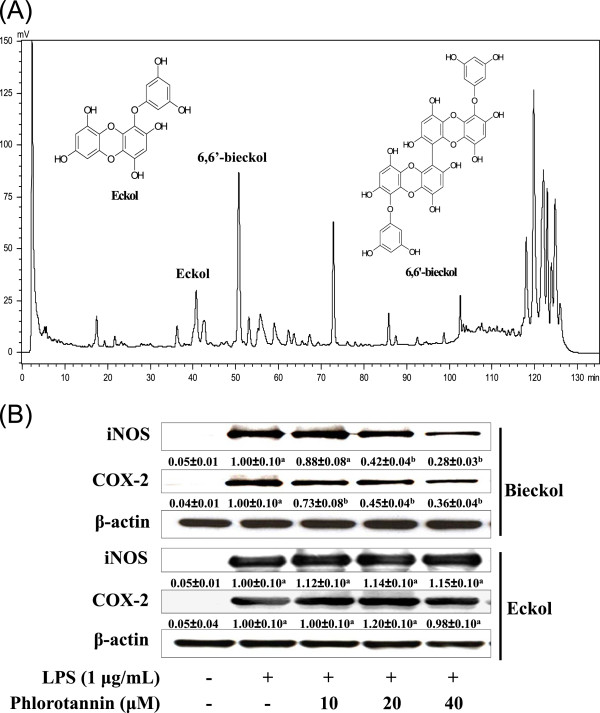
**Identification of eckol and 6,6’-bieckol in EMM.** (**A**) HPLC chromatogram of EMM and the chemical structures of eckol and 6,6’-bieckol (insert). (**B**) Cells pretreated with different concentrations of eckol or 6,6’-bieckol for 1 h were stimulated with LPS for 16 h. The expression levels of iNOS and COX-2 in cells were analyzed by Western blotting using corresponding antibodies. Relative density ratios of each protein over β-actin are shown below the blots. The results presented are representative of three independent experiments. ^a^*p* < 0.05 indicates significant differences compared to the control group. ^b^*p* < 0.05 indicates significant differences compared to the LPS-only treated group.

EMM was further separated by reverse-phase column chromatography (Figure 8A) for the isolation of anti-inflammatory compound(s). The chemical structures of the isolated compounds were identified as eckol and 6,6’-bieckol, which were isolated for the first time from *M. myagroides,* from the comparison of their NMR spectra with the published spectral data. We obtained 2.5 mg of eckol and 5.6 mg of 6,6’-bieckol from 1 g of EMM. The purified 6,6’-bieckol significantly inhibited LPS-induced iNOS and COX-2 production in a dose-dependent manner (*p* < 0.05, Figure 8B), however, eckol did not affect on the production of inflammatory proteins. Furthermore, pre-administration of 6,6'-bieckol (30 μg/ear) markedly inhibited the PMA-induced ear edema with 64.1% inhibition (Figure 7, *p* < 0.05). The result indicates that 6,6’-bieckol is one of the anti-inflammatory compounds in EMM.

## Discussion

The present study was undertaken to examine the anti-inflammatory effect of EMM on LPS-stimulated murine macrophage cells. To further understood the molecular mechanisms of EMM, we investigated the effects of EMM on the secretion of NO, PGE_2_, TNF-α, IL-1β, and IL-6, the expression of iNOS and COX-2, and the activation of NF-κB. Our results indicated that EMM effectively inhibited the secretion of NO, PGE_2_, TNF-α, IL-1β, and IL-6 through a blockade of the NF-κB and MAPK pathways in LPS-stimulated macrophages. The inhibitory effect of EMM on the expression of inflammatory mediators suggested one of the mechanisms responsible for its anti-inflammatory action and its potential for use as a therapeutic agent for treating inflammatory diseases.

Under pathological conditions, excessive inflammatory mediators and pro-inflammatory cytokines produced by activated macrophages cause inflammatory process and act synergistically with other inflammatory mediators [2,4,18]. Compounds able to reduce NO or PGE_2_ production may be attractive as anti-inflammatory agents and, for this reason, the inhibitory effects of natural compounds on NO or PGE_2_ productions have been rigorously studied to develop therapeutic agent against inflammatory diseases [17,19,20]. Also, excessive production of pro-inflammatory cytokines plays a critical role in acute inflammatory responses as well as chronic inflammatory diseases. Recent studies have shown that *in vivo* or *in vitro* treatments of natural compounds are effective in reducing inflammation by the suppression of pro-inflammatory cytokines, which may ameliorate inflammation-related diseases, including atherosclerosis, cancer, and inflammatory arthritis [21,22]. Thus, the regulation of those molecules is important to the inhibition of inflammatory response. Our results indicate that the inhibition of NO and PGE_2_ production by EMM in LPS-stimulated macrophage cells is associated with downregulation of iNOS and COX-2 genes (Figure 2), which seems the first addressing transcriptional inhibition of iNOS and COX-2 by EMM.

NF-κB is a transcription factor actively involved in the transcriptional induction of iNOS and COX-2 gene [1,3]. Like NO and PGE_2_ produced by iNOS and COX-2, respectively, the release of pro-inflammatory cytokines is regulated by NF-κB pathway and plays an important role in the induction of the innate immune response of the acquired immune response [23]. It has been well studied that the inhibition of NF-κB activation by black tea extract is associated with the phosphorylation, ubiquitination, and subsequent degradation of IκB via ubiquitin-proteosome pathway [24]. Although biochemical actions of EMM on NF-κB regulations remain unknown, the present study showed that EMM potentially inhibits the proteolytic degradation of IκB-α and the NF-κB promoter-driven luciferase expression induced by LPS in RAW 264.7 cells. Therefore, these results demonstrate the ability of EMM to inhibit NF-κB activation in response to LPS signal in RAW 264.7 cells. Hence it is likely that reductions of iNOS, COX-2, and pro-inflammatory cytokine expression in RAW 264.7 cells are largely associated with activation/deactivation of NF-κB pathway. This report is, to our best knowledge, the novel findings to address the functions of EMM through NF-κB pathway in response to LPS treatment.

NF-κB is also regulated by various signaling kinases including MAPKs (ERK, JNK, and p38) and Akt, which are groups of signaling molecules to play key roles in NF-κB activation [2,3]. MAPKs have been suggested to be involved in pro-inflammatory signaling cascades and in the activation of NF-κB in LPS-stimulated immune cells [2,11]. Therefore, anti-inflammatory mechanisms are closely related with inhibition of MAPKs in activated RAW 264.7 cells. In this study, we found that phosphorylation of MAPKs in response to LPS was inhibited by EMM treatment (Figure 6A). Interesting finding of this study is that the activation of Akt, a downstream regulator of PI3K, was also inhibited by EMM in response to LPS signal in RAW 264.7 cells. Precise mechanisms by which EMM modulate this PI3K/Akt pathway in the LPS stimulation remains unclear. Considering a recent study with a phlorofucofuroeckol A, which indicated a link between ROS and PI3K/Akt pathway in regulations of inflammatory genes [7,11], it is speculated that the antioxidant activity of EMM or unknown components in EMM may be related to inhibition of Akt phosphorylation. Thus, it is likely that inhibition of MAPKs and Akt phosphorylation by EMM may contribute to the EMM-mediated inhibition of NF-κB pathway in LPS-stimulated RAW 264.7 cells.

Topical application of PMA to mouse skin induces inflammatory response and this is a well-established *in vivo* model for the evaluation of various anti-inflammatory agents. PMA can activate a wide variety of cell types that may contribute to acute inflammation, resulting in an increase in epidermal tissue swelling and infiltration of inflammatory cells [25,26]. The results from our *in vivo* experiments clearly showed that EMM (90 μg/ear) suppressed 66.8% of PMA-induced skin swelling. Furthermore, 6,6’-bieckol (30 μg/ear) isolated firstly from *M. myagroides* in this study inhibited 64.1% of PMA-induced skin swelling, indicating that it might be a main anti-inflammatory compound in EMM. Although the inhibitory activity of EMM is about one third of 6,6’-bieckol, EMM showed higher anti-edema activity than Indo compared to its concentration. This result demonstrates that EMM can be used as a topical anti-inflammatory agent.

Like other polyphenolic compounds, phlorotannins, a class of compounds polymerized with phloroglucinol units found in brown seaweeds, have strong antioxidant [18,27] and anti-inflammatory [11,28] activities. The multifunctional antioxidant activity of phlorotannins is highly associated with phenol rings which act as electron traps to scavenge peroxy, superoxide-anions, and hydroxyl radicals [29]. Specific mechanisms of phlorotannins on the anti-inflammatory actions are not clearly defined. Considering cellular signaling of polyphenols, phlorotannins may not merely exert their effects as free radical scavenger, but may also modulate inflammatory cellular signaling proteins, including NF-κB and AP-1 transcription factors [30]. Recent studies demonstrated that phlorofucofuroeckol A and 6,6’-bieckol isolated from *Ecklonia* spp. alleviate inflammatory response to LPS by inhibiting NF-κB pathway with its intrinsic antioxidant activity [11,28]. In the present study, the isolated 6,6’-bieckol from EMM remarkably inhibited the production of iNOS and COX-2 proteins, indicating 6,6’-bieckol is one of the principle anti-inflammatory compounds in EMM.

## Conclusions

We demonstrated that EMM inhibited the secretion of inflammatory mediators, such as NO and PGE_2_, and pro-inflammatory cytokines, including TNF-α, IL-1β, and IL-6, in LPS-stimulated RAW 264.7 macrophages. Moreover, the inhibitory effect of EMM was associated with inactivation of the NF-κB pathway via blocking the phosphorylation of MAPKs and Akt. In addition, the results of *in vivo* study showed that EMM can be applicable to a topical anti-inflammatory agent.

## Abbreviations

COX-2: Cyclooxygenase 2; DAPI: 4’,6-Diamino-2-phenylindole; DMEM: Dulbecco’s modified Eagle’s medium; DMSO: Dimethyl sulfoxide; EMM: Ethanolic extract of *Myagropsis myagroides*; ERK: Extracellular signal regulated kinase; ELISA: Enzyme-linked immunosorbent assay; GAPDH: Glyceraldehyde 3-phosphate dehydrogenase; HPLC: High-performance liquid chromatography; IκB: Inhibitor of κB; IKK: IkappaB kinase; IL: Interleukin; iNOS: Inducible nitric oxide synthase; JNK: c-Jun N-terminal kinase; LPS: Lipopolysaccharide; MAPK: Mitogen-activated protein kinase; NMR: Nuclear magnetic resonance; NF-κB: Nuclear factor-κB; NO: Nitric oxide; PBS: Phosphate-buffered saline; PGE_2_: Prostaglandin E_2_; PMSF: Phenylmethylsulfonyl fluoride; PCR: Polymerase chain reaction; TNF-α: Tumor necrosis factor-α; PMA: Phorbol 12-myristate 13-acetate.

## Competing interests

The authors declare that they have no competing interests.

## Authors’ contributions

E-JJ carried out the main experiment and wrote the manuscript. M-SL prepared EMM and performed partial Western blotting. J-WC performed immunocytochemistry. JSK performed ELISA for measuring the proinflammatory cytokines. TS and B-MJ isolated and identified anti-inflammatory compounds from EMM. NYY and C-WL harvested *M. myogroides*. J-IK contributed to discuss and write the manuscript. H-RK designed and organized this study. All authors read and approved the final version of the manuscript.

## Pre-publication history

The pre-publication history for this paper can be accessed here:

http://www.biomedcentral.com/1472-6882/12/171/prepub

## References

[B1] Xie QW Whisnant R Nathan C Promoter of the mouse gene encoding calcium-independent nitric oxide synthase confers inducibility by interferon γ and bacterial lipopolysaccharide J Exp Med 1993 177 1779 1784 10.1084/jem.177.6.1779 7684434 PMC2191051

[B2] Zhang G Ghosh S Molecular mechanisms of NF-κB activation induced by bacterial lipopolysaccharide through Toll-like receptors J Endotoxin Res 2000 6 453 457 11521070 10.1179/096805100101532414

[B3] Marks-Konczalik J Chu SC Moss J Cytokine-mediated transcriptional induction of the human inducible nitric oxide synthase gene requires both activator protein 1 and nuclear factor κB-binding sites J Biol Chem 1998 273 22201 22208 10.1074/jbc.273.35.22201 9712833

[B4] Vane JR Mitchell JA Appleton I TomLinson A Bishop-Bailey D Croxtall J Willoughby DA Inducible isoforms of cyclooxygenase and nitric-oxide synthase in inflammation Proc Natl Acad Sci U S A 1994 91 2046 2050 10.1073/pnas.91.6.2046 7510883 PMC43306

[B5] Makarov SS NF-κB in rheumatoid arthritis: a pivotal regulator of inflammation, hyperplasia, and tissue destruction Arthritis Res Ther 2001 3 200 206 10.1186/ar300 PMC128895 11438035

[B6] Guha M Mackman N LPS induction of gene expression in human monocytes Cell Signal 2001 13 85 94 10.1016/S0898-6568(00)00149-2 11257452

[B7] Kao SJ Lei HC Kuo CT Chang MS Chen BC Chang YC Chiu WT Lin CH Lipoteichoic acid induces nuclear factor-κB activation and nitric oxide synthase expression via phosphatidylinositol 3-kinase, Akt, and p38 MAPK in RAW 264.7 macrophages Immunology 2005 115 366 374 10.1111/j.1365-2567.2005.02160.x 15946254 PMC1782163

[B8] Zou Y Qian ZJ Li Y Kim MM Lee SH Kim SK Antioxidant effects of phlorotannins isolated from Ishige okamurae in free radical mediated oxidative systems J Agric Food Chem 2008 56 7001 7009 10.1021/jf801133h 18616277

[B9] Okada Y Ishimaru A Suzuki R Okuyama T A new phloroglucinol derivative from the brown alga Eisenia bicyclis: potential for the effective treatment of diabetic complications J Nat Prod 2004 67 103 105 10.1021/np030323j 14738398

[B10] Yoon NY Chung HY Kim HR Choi JS Acetyl- and butyrylcholinesterase inhibitory activities of sterols and phlorotannins from Ecklonia stolonifera Fish Sci 2008 74 200 207 10.1111/j.1444-2906.2007.01511.x

[B11] Kim AR Lee MS Shin TS Hua H Jang BC Choi JS Byun DS Utsuki T Ingram D Kim HR Phlorofucofuroeckol A inhibits the LPS-stimulated iNOS and COX-2 expressions in macrophagesviainhibition of NF-κB, Akt, and p38 MAPK. Toxicol In Vitro 2011 25 1789 1795 10.1016/j.tiv.2011.09.012 21963823

[B12] Lee MS Shin TS Utsuki T Choi JS Byun DS Kim HR Isolation and identification of phlorotannins from Ecklonia stolonifera with antioxidant and epatoprotective properties in tacrine-treated HepG2 cells J Agric Food Chem 2012 60 5340 5349 10.1021/jf300157w 22587607

[B13] Wong CK Ooi VE Ang PO Protective effects of seaweeds against liver injury caused by carbon tetrachloride in rats Chemosphere 2000 41 173 176 10.1016/S0045-6535(99)00407-5 10819197

[B14] Heo SJ Yoon WJ Kim KN Ahn GN Kang SM Kang DH Affan A Oh C Jung WK Jeon YJ Evaluation of anti-inflammatory effect of fucoxanthin isolated from brown algae in lipopolysaccharide-stimulated RAW 264.7 macrophages Food Chem Toxicol 2010 48 2045 2051 10.1016/j.fct.2010.05.003 20457205

[B15] Kim H Lee HS Chang KT Ko TH Baek KJ Kwon NS Chloromethyl ketones block induction of nitric oxide synthase in murine macrophages by preventing activation of nuclear factor-κB J Immunol 1995 154 4741 4748 7536778

[B16] Lee JY Lee MS Choi HJ Choi JW Shin T Woo HC Kim JI Kim HR Hexane fraction fromLaminaria japonicaexerts anti-inflammatory effects on lipopolysaccharide-stimulated RAW 264.7 macrophages via inhibiting NF-kappaB pathway. Eur J Nutr 2012 [Epub ahead of print] 10.1007/s00394-012-0345-1 22476925

[B17] Kim AR Shin TS Lee MS Park JY Park KE Yoon NY Kim JS Choi JS Jang BC Byun DS Park NK Kim HR Isolation and identification of phlorotannins from Ecklonia stolonifera with antioxidant and anti-inflammatory properties J Agric Food Chem 2009 57 3483 3489 10.1021/jf900820x 19338274

[B18] Libby P Inflammation and cardiovascular disease mechanisms Am J Clin Nutr 2006 83 456S 460S 16470012 10.1093/ajcn/83.2.456S

[B19] Gupta M Mazumder UK Gomathi P Selvan VT Antiinflammatory evaluation of leaves of Plumeria acuminata BMC Complement Altern Med 2006 6 36 10.1186/1472-6882-6-36 17081283 PMC1654182

[B20] Elmann A Mordechay S Erlank H Telerman A Rindner M Ofir R Anti-neuroinflammatory effects of the extract of Achillea fragrantissima BMC Complement Altern Med 2011 11 98 10.1186/1472-6882-11-98 22018032 PMC3213061

[B21] Garcia-Lafuente A Guillamon E Villares A Rostagno MA Martinez JA Flavonoids as anti-inflammatory agents: implications in cancer and cardiovascular disease Inflamm Res 2009 58 537 552 10.1007/s00011-009-0037-3 19381780

[B22] Ekambaram S Perumal SS Subramanian V Evaluation of antiarthritic activity of Strychnos potatorum Linn seeds in Freund's adjuvant induced arthritic rat model BMC Complement Altern Med 2010 10 56 10.1186/1472-6882-10-56 20939932 PMC2978115

[B23] Netea MG Warris A Van der Meer JW Fenton MJ Verver-Janssen TJ Jacobs LE Andresen T Verweij PE Kullberg BJ Aspergillus fumigatus evades immune recognition during germination through loss of toll-like receptor-4-mediated signal transduction J Infect Dis 2003 188 320 326 10.1086/376456 12854089

[B24] Song YA Park YL Kim KY Chung CY Lee GH Cho DH Ki HS Park KJ Cho SB Lee WS Kim N Ahn BW Joo YE Black tea extract prevents lipopolysaccharide-induced NF-κB signaling and attenuates dextran sulfate sodium-induced experimental colitis BMC Complement Altern Med 2011 11 91 10.1186/1472-6882-11-91 21989142 PMC3207919

[B25] Wershil BK Murakami T Galli SJ Mast cell-dependent amplification of an immunologically nonspecific inflammatory response. Mast cells are required for the full expression of cutaneous acute inflammation induced by phorbol 12-myristate 13-acetate J Immunol 1988 140 2356 2360 3280681

[B26] Garrido G Gonzalez D Lemus Y Garcia D Lodeiro L Quintero G Delporte C Nunez-Selles AJ Delgado R In vivo and in vitro anti-inflammatory activity of Mangifera indica L. extract (VIMANGR®) Pharmacol Res 2004 50 143 149 10.1016/j.phrs.2003.12.003 15177302

[B27] Kang HS Chung HY Kim JY Son BW Jung HA Choi JS Inhibitory phlorotannins from the edible brown alga Ecklonia stolonifera on total reactive oxygen species (ROS) generation Arch Pharm Res 2004 27 194 198 10.1007/BF02980106 15022722

[B28] Yang YI Shin HC Kim SH Park WY Lee KT Choi JH 6,6'-Bieckol, isolated from marine alga Ecklonia cava, suppressed LPS-induced nitric oxide and PGE2 production and inflammatory cytokine expression in macrophages: The inhibition of NF-κB Int Immunopharmacol 2012 12 510 517 10.1016/j.intimp.2012.01.005 22289571

[B29] Wang T Jónsdóttir R Ólafsdóttir G Total phenolic compounds, radical scavenging and metal chelation of extracts from Icelandic seaweeds Food Chem 2009 116 240 248 10.1016/j.foodchem.2009.02.041

[B30] Rahman I Biswas SK Kirkham PA Regulation of inflammation and redox signaling by dietary polyphenols Biochem Pharmacol 2006 72 1439 1452 10.1016/j.bcp.2006.07.004 16920072

